# Exercises on Chapter Third

**Published:** 1883-09

**Authors:** 


### EXERCISES ON CHAPTER THIRD.

What can be said of the physical condition of the elements?

Of the thirty-nine having pharmaceutical interest, how many are solids?

How many are liquids?

How many are gaseous?

What is the more general classification of the elementary bodies?

How many are metals?

How many are non-metals?

What metal is a liquid?

What non-metal is a liquid?

Give the names of all the solid elements?

Are the gases metals or non-metals?

Name the elementary gases?

What step is first in order to the study of the gases?

What apparatus are requisite for the generation and collection of oxygen?

What is the usual source of oxygen?

Describe the method of preparing it?

Give the history of oxygen?

What name was given it by Dr. Priestley?

Why did he so name it?

Who gave it its present name?

On what erroneous view did he call it oxygen?

Did Priestley live to know the nature of oxygen and the importance of its discovery?

Is oxygen chiefly concerned in the formation of acids?

What element is?

Is oxygen an abundant element?

How much of the earth's bulk is oxygen?

How much of the volume of the atmosphere is oxygen?

How much of the weight of all waters is oxygen?

What part of all animal matter is oxygen?

What part of all vegetables?

Give the chemical properties of oxygen?

Give its physical properties?

Is it heavier or lighter than air?

Hydrogen being 1, what is the weight of oxygen?

What is the difference between the specific gravity of a gas and its density?

What is the quantivalence of oxygen?

Is the quantivalence of an element a chemical or a physical property?

What is the electro-chemical condition of oxygen?

In the preparation of oxygen did the manganese dioxide undergo any change?

Why was it used then?

By what name is this curious action of the manganese dioxide known?

What is the usual source of hydrogen, and how is it prepared?

What is the danger of igniting hydrogen when mixed with air?

Give the chemical properties of hydrogen?

What is the number of its heat units?

Why will an animal die when breathing nothing but hydrogen?

Give the physical properties of hydrogen?

What is its specific gravity?

Of what is it the physical standard?

Of what is it the chemical standard?

What is the weight of a liter of hydrogen?

